# Human pulmonary artery endothelial cells upregulate ACE2 expression in response to iron‐regulatory elements: Potential implications for SARS‐CoV‐2 infection

**DOI:** 10.1002/pul2.12068

**Published:** 2022-04-08

**Authors:** Quezia K. Toe, Theo Issitt, Abdul Mahomed, Fatma Almaghlougth, Ishan Bahree, Charles Sturge, Xueqi Hu, Ioannis Panselinas, Anne Burke‐Gaffney, Stephen, Jc Wort, Gregory John Quinlan

**Affiliations:** ^1^ Cario‐Respiratory Interface, National Heart Lung Institute Imperial College London London UK; ^2^ Biology Department University of York York UK

**Keywords:** COVID‐19, hepcidin/ferroportin axis, interleukin‐6, pulmonary artery endothelial cells

## Abstract

Vascular endothelial cell dysfunction is reported in severe coronavirus disease 2019 disease, however, controversy exists regarding levels of angiotensin‐converting enzyme 2 (ACE2) expression, a coreceptor for severe acute respiratory syndrome coronavirus 2, in these cells. We report ACE2 expression and positive regulation by both interleuki‐6, hepcidin, and ferroportin knock‐down in pulmonary artery endothelial cells with potential implications for viral infection.

## INTRODUCTION

The current coronavirus disease 2019 (COVID‐19) pandemic has resulted in significant global impacts on healthcare systems and beyond. Characterized as a respiratory infection, which for the majority of those infected is minor or asymptomatic, there are nevertheless significant life‐threatening complications for a proportion of individuals. Age and or co‐morbidities which are often chronic in nature are the main risk factors for severe COVID‐19 infection with strong further associations with mortality. Severe acute respiratory distress syndrome (ARDS) is the primary disease presentation for those requiring intensive care treatment including the need for supportive ventilation or, in extreme cases, extracorporeal membrane oxygenation (ECMO). Whilst respiratory failure is a principal mortality factor for COVID‐19, emerging data indicate that other organ failures including the kidney and cardiovascular system contribute to death. Evidence of widespread thrombosis with micro‐thrombosis seen in the vasculature of lungs, kidneys, and brain, and other organs together with the presence of biomarkers of vascular dysfunction in blood samples from severe COVID‐19 patients indicate a primary role for the endothelium in the disease process. It is recognized that severe acute respiratory syndrome coronavirus 2 (SARS‐CoV‐2) primarily infects cells by using a viral surface glycoprotein (spike protein) which binds to angiotensin‐converting enzyme related carboxypeptidase (ACE2) on the target cell to gain entry. ACE2 is reported to be expressed on many cell types including lung epithelial and vascular endothelium as reported in the previous SARS‐CoV‐1 epidemic.^1^ However, current research efforts are unable to demonstrate any or very low levels of ACE2 expression in human endothelial cells,^2^ which poses a question as to how SARS‐CoV‐2 can infect and cause endotheliitis as widely reported.

**Figure 1:**
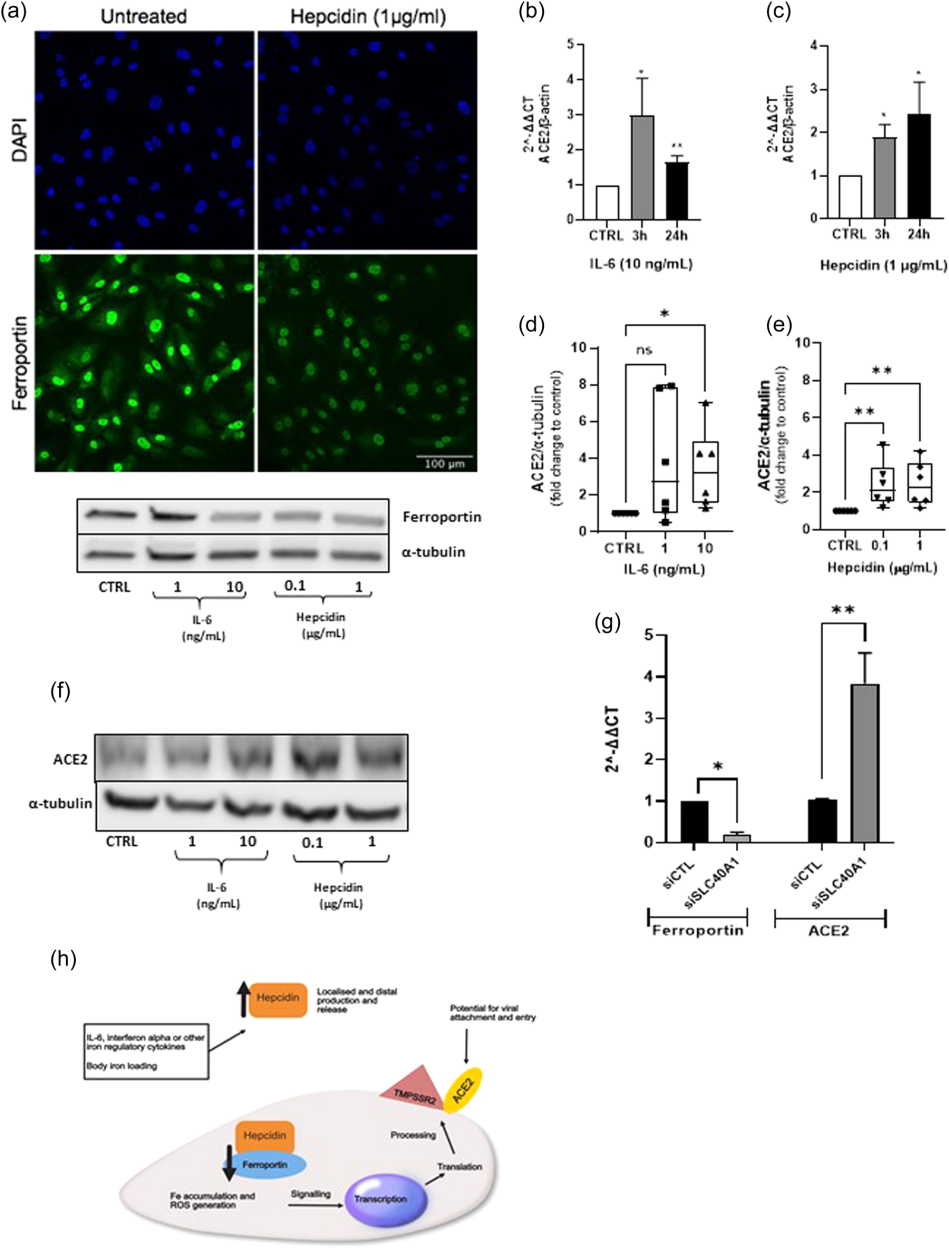
Hepcidin and IL‐6 upregulate ACE‐2 expression in hPAECs. (A) Ferroportin is expressed by hPAECs and modulated by IL‐6 and hepcidin. PAECs were treated by media alone, IL‐6 (1 and 10 ng/ml) and hepcidin (0.1 and 1 μg/ml) for 24 h, cells were lysed, and 50 μg of protein separated on 4%–15% SDS‐page and transferred onto nitrocellulose membranes. Western blot analysis was performed using rabbit anti‐Ferroportin (1:1000) and α‐tubulin (1:2000). (B and C) ACE2 mRNA transcription was upregulated by IL‐6 and hepcidin. RT‐PCR was performed using SYBR green (Bioline) with human ACE2 primers and β‐actin, after hPAECs were treated with media alone, IL‐6 (10 ng/ml) or hepcidin (μg/ml) for 3 or 24 h. The values were normalized as fold changes to the control of untreated cells at each corresponding time point. *N* = 3, student's *t* test was performed; *p < 0.05, **p < 0.01. (D–F) IL‐6 and hepcidin increased ACE2 protein expression. PAECs were treated by media alone, IL‐6 (1 and 10 ng/ml) and hepcidin (0.1 and 1 μg/ml) for 24 h, cells were lysed, and 50 μg of protein separated on 4%–15% SDS‐PAGE and transferred onto nitrocellulose membranes. Western blot analysis was performed using rabbit anti‐ACE2 (1:500) and α‐tubulin (1:2000). Relative protein expression quantification was performed with ImageJ software. Values were normalized to α‐tubulin and fold change calculated against control. Three PAECs donors were used, two experiments were run for each donor. One‐way ANOVA nonparametric Kruskal–Wallis test was performed with post hoc Dunn's test for multiple testing correction; *p < 0.05, **p < 0.01. (F) Ferroportin knockdown upregulates ACE2 expression. hPAECs were incubated with siSLC40A1 (to silence ferroportin) and a nontargeting siRNA (negatve control) for 6 h, RNA was then isolated after 48 h. RT‐PCR was performed using SYBR green (Bioline) with human ACE2, Ferroportin, and β‐actin primers. The values were normalized as fold changes to the control of untreated cells at each corresponding time point. *N* = 3, Student's *t* test was performed; *p < 0.05, **p < 0.01. (H) Elevated levels of hepcidin, which is positively regulated by increased in body iron levels and or iron regulatory cytokines including IL‐6, bind to the cellular iron export protein ferroportin on PAECs. Internalization of ferroportin instituted by hepcidin binding results in cellular iron accumulation altering the intracellular environment with potential impacts on reactive oxygen species generation and organelle functions. Resultant cellular changes, signal ACE2 gene transcription, and protein expression. ACE‐2, angiotensin‐converting enzyme 2; IL‐6, interleukin‐6; hPAECs, human pulmonary artery endothelial cells; mRNA, messenger RNA; RT‐PCR, real‐time polymerase chain reaction; SDS‐PAGE, sodium dodecyl sulfate‐polyacrylamide gel electrophoresis; siRNA, small interfering RNA

Interleukin‐6 (IL‐6) is reported to be greatly elevated in patients with severe COVID‐19 infection.^3^ As a key positive regulator for hepcidin biosynthesis this may suggest impacts on iron metabolism in these patients; elevated serum ferritin further reinforces this notion. Hepcidin, described as the global regulator for iron homeostasis, is released from the liver and binds to the cellular iron exporter, ferroportin, causing internalization; thereby, preventing cellular iron export. The axis is operational in the liver, gut, and cells chiefly involved in iron recycling for processes including erythropoiesis (reviewed by Reichert et al.).^4^ Our studies have identified a localized hepcidin/ferroportin axis that is operational in human pulmonary artery smooth muscle cells (hPASMCs); importantly, IL‐6 promotes hepcidin production by hPASMCs.^5^ We now report the presence of this axis in human pulmonary artery endothelial cells (hPAECs) (Figure 1A), which shows ferroportin expression and downregulation by hepcidin (confocal images) together with a western blot which further demonstrates IL‐6 mediated downregulation. These observations prompted us to consider a role for IL‐6 and the hepcidin/ferroportin (H/F) axis as a modular for ACE2 expression in human pulmonary artery endothelial cells (hPAECs), given that interactions between ACE2 and IL‐6 have previously been described.^6^ We hypothesize that iron retention by PAECs which is caused by hepcidin mediated inhibition of ferroportin iron export, signals ACE2 transcription and protein expression; IL‐6 may further contribute via autocrine hepcidin production (see also Figure 1H).

## METHODS

Studies were undertaken with hPAECs obtained from ATCC®. hPAECs were grown according to supplier's instructions.

### Cell treatments

hPAECs cells were treated by media alone, IL‐6 (1 and 10 ng/ml) and hepcidin (0.1 and 1 μg/ml) and harvested at different time points depending on the incubation time required for each experiment.

### Immuno‐fluorescence

Following 24 h treatments, Cells were then fixed using 4% paraformaldehyde, permeablized with 0.2% triton x‐100, blocked in 10% bovine serum albumin, and incubated with rabbit anti‐ferroportin (1:1000), washed with phosphate‐buffered saline three times, incubated with Alexaflour anti‐rabbit 700 (1:500), counterstained with 4′,6‐diamidino‐2‐phenylindole and visualized. Images show maximum intensity per pixel across the Z plane.

### Real‐time polymerase chain reaction (RT‐PCR)

RT‐PCR was performed using SensiFast SYBR green Lo‐ROX (Meridian Biosciences) with human ACE2 primers (forward – GTTTGTAACCCAGATAATCCAC; reverse – AATGATTTGCTCTTGCCATC) and β‐actin (forward – GACGACATGGAGAAAATCTG; reverse – ATGATCTGGGTCATCTTCTC), after hPAECs were treated for 3 or 24 h and complementary DNA transcription. The values were normalized as fold changes to the control of untreated cells at each corresponding time point.

### Western blot

hPAECs were lysed with radioimmunoprecipitation assay buffer (supplemented with protease and phosphatase inhibitors) after 24 h treatments with IL‐6 and hepcidin. Bradford assay was performed to quantify protein and 50 μg of protein was separated on 4%–15% sodium dodecyl sulfate‐polyacrylamide gel electrophoresis and transferred onto nitrocellulose membranes. Western blot analysis was performed using rabbit anti‐Ferroportin (1:1000; NBP1‐21502, NOVUS), rabbit anti‐ACE2 (1:500; ab15348, Abcam), and α‐tubulin (1:2000; 3873S, Cell Signalling). Membranes were imaged with the Odyssey FC imaging system.

### Gene knockdown

In brief, hPAECs cells were seeded in a sox‐well plate at 70% confluence. Once cells were completely attached they were incubated with 20 nM of ferroportin specific small interfering RNA (siRNA) and 20 nM of nontargeting siRNA oligonucleotides (negative control) for 6 h. After the incubation with siRNAs fresh media was added and cell lysates were collected at different time points for mRNA analysis.

## RESULTS

When challenged with either IL‐6 or hepcidin, significant upregulation of ACE2 mRNA over time was observed with hPAECs from 3 donors (Figure 1B,C). Importantly, significantly elevated levels of ACE2 protein expression were also observed over time by western blot (Figure 1C,D); Figure 1F shows a typical western blot image (full blots image Supporting Information Figure). In addition, knockdown of the ferroportin gene (SLC40A1) in these cells resulted in a significant loss of ferroportin mRNA coupled with a strong significant upregulation of ACE2 mRNA (Figure 1G), a result which further supports a role for the modulation of the hepcidin/ferroportin axis as a regulator for ACE2 expression in hPAECs. We were also able to demonstrate obvious basal expression (mRNA and protein) for TMPRSS2, another essential coreceptor for viral entry into cells, but this was not subject to modulation by the treatments used (data not shown). Given the potential pulmonary vascular specific context of these findings and the known importance of shear stress as a modulator of gene expression for hPAECs in vivo, an assessment of ACE2 transcript expression was undertaken by subjecting hPAECs to laminar flow (15 dyn/cm^2^) in a parallel‐plate fluid flow chamber for 10 h. While, ACE2 did not prove to be a flow responsive gene (data not shown), more extensive studies investigating variable flow states need to be conducted to address different forms of flow, exposure, and other variables before firm conclusions can be drawn.

Taken together these observations may provide an explanation for viral vascular endothelial cell infection seemly seen in severe COVID‐19 disease, at least in a vascular setting.

## DISCUSSION

Elevated levels of hepcidin have been linked to age‐associated inflammation^7^ and anemia.^8^ and similar effects are seen in chronic/comorbid disease settings (anemia of chronic disease).^9^ Given our findings, it is not unreasonable to speculate that aging when associated with inflammation/anemia and chronic disease settings may predispose these groups to more severe vascular disease because of the greater likelihood of elevated ACE2 expression linked to ongoing elevated hepcidin levels and particularly in pulmonary vascular beds. The potential localized nature of these responses is further reinforced by studies undertaken using human aortic endothelial cells. Limited presence of ACE2 mRNA and protein was observed in these cells and attempts to modulate the H/F axis including the use of IL‐6 did not affect ACE2 expression (data no not shown), failing to replicate hPAEC studies.

Emerging literature is also establishing links between the hepcidin/ferroportin axis and SARS‐CoV‐2^10^ with some sequence homology between the SARS‐CoV‐2 spike protein and hepcidin reported.^11^ Interestingly, CD26 another suggested coreceptor for SARS‐CoV‐2 is also known to bind to ferroportin.^12^ Moreover, published studies from China and Italy^13^, ^14^ have reported strong associations with the severity of disease and mortality and hepcidin levels. The significance of these findings remains to be evaluated but does further highlight a potential link between dysregulated iron metabolism and COVID‐19 infection. IL‐6, a positive regulator of hepcidin, also upregulated ACE2 expression in hPAECs. A recent study^15^ has suggested that interferon‐α is the main regulator for ACE2 in PAECs and lung microvascular endothelial cells with much lesser impacts observed with IL‐6. Examination of the methodology used by these researchers differs somewhat from our own and was confined to mRNA analysis alone and at a single time point. Interestingly, interferon‐α is a positive regulator for hepcidin production.^16^

Whilst our studies demonstrate upregulation of ACE2 in human hPAECs in response to hepcidin or IL‐6, studies need to be undertaken to establish if such effects result in enhanced SARS‐CoV‐2 infection. Should this be the case, targeting the H/F axis may well provide therapeutic benefit in severe COVID‐19 infection and further supports the use of the IL‐6 receptor antibody, tocilizumab.^17^

## AUTHOR CONTRIBUTIONS

Quezia Keller Toe, Theo Issitt, Stephen John Wort, and Gregory John Quinlan contributed to the study concept and design. Quezia Keller Toe, Theo Issitt, Abdul Mahomed, Fatma Almaghlougth, Ishan Bahree, Charles Sturge, Xueqi Hu, and Ioannis Panselinas contributed to the acquisition and analysis of data. Gregory John Quinlan, Stephen John Wort, Anne Burke‐Gaffney, and Quezia Keller Toe wrote the manuscript.

## ETHICS STATEMENT

Ethics statement is not applicable to this study as commercially available Pulmoary artery enodthelial cells were used throughout.

## Supporting information

Supporting information.

Supporting information.
