# Supplementary figures and images for: Human pulmonary artery endothelial cells upregulate ACE2 expression in response to iron‐regulatory elements: Potential implications for SARS‐CoV‐2 infection
Source: Pulm Circ. 2022 Apr 8;12(2):e12068. doi: 10.1002/pul2.12068 (PMC9063967; doi:10.1002/pul2.12068)

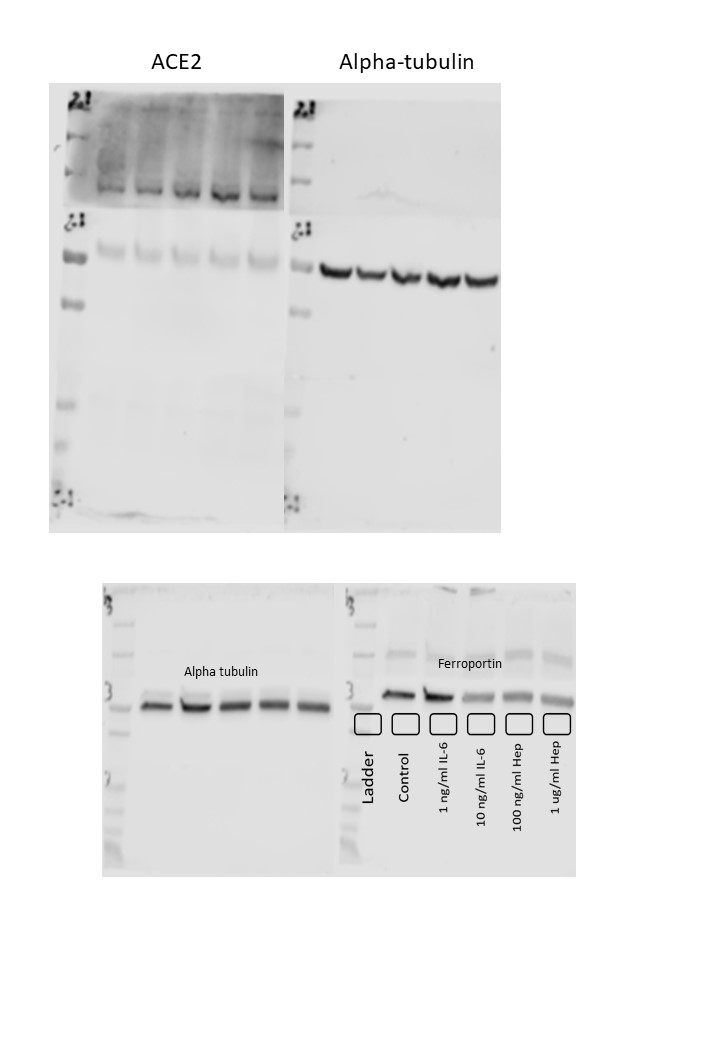

Supplement: Supplementary file 1 — Supporting information. [file PUL2-12-0-s001.jpg]
