# Supplementary material for: Human pulmonary artery endothelial cells upregulate ACE2 expression in response to iron‐regulatory elements: Potential implications for SARS‐CoV‐2 infection
Source: Pulm Circ. 2022 Apr 8;12(2):e12068. doi: 10.1002/pul2.12068 (PMC9063967; doi:10.1002/pul2.12068)
Supplement: Supplementary file 2 — Supporting information. [file PUL2-12-0-s002.docx]

Supplementary figure – Full western blots (WB) representations of the WBs presented in the main paper are shown. The topmost image shows ACE2 protein (left) and alpha-tubulin (right). The image below shows alpha-tubulin (left) and ferroportin (right). Sample loading for all images was prescribed according to the ferroportin image (bottom, right).
